# Trace Radiocarbon Analysis of Environmental Samples

**DOI:** 10.6028/jres.093.048

**Published:** 1988-06-01

**Authors:** Ann E. Sheffield, Lloyd A. Currie, George A. Klouda

**Affiliations:** National Bureau of Standards, Center for Analytical Chemistry, Gas and Particulate Science Division, Gaithersburg, MD 20899

## 1. Radiocarbon in the Environment

In the atmosphere, radioactive ^14^C is produced at a roughly constant rate and decays with a half-life of 5730 y. Human activity perturbs the natural, steady-state level of ^14^C. Combustion of fossil fuels and emission of pollutants derived from fossil feedstocks dilute ^14^C with “dead” ^12^C (the “Suess effect”), and nuclear weapons tests produce excess ^14^C (the “bomb effect”). Therefore, sources are often revealed by measurement of ^14^C in species of environmental concern, such as “greenhouse” gases (CO_2_, CO, CH_4_), atmospheric particles, and toxic or carcinogenic organic compounds.

Atmospheric weapons testing peaked in the early 1960s, and, in 1963, the atmospheric radiocarbon level was about twice the natural level [1]. Since then, the ^14^C concentration has been declining, largely because of equilibration of atmospheric CO_2_ with carbonate in the world’s oceans. The changing distribution of “bomb” carbon in the ocean has been measured to investigate ocean circulation patterns [2,3,4]. As nuclear power plants become more widely used, they, too, may become important sources of atmospheric ^14^C. Radiocarbon has already been exploited as an inherent tracer to investigate the dispersion of plumes from nuclear power plants [5,6,7].

## 2. Accelerator Mass Spectrometry

For many species of interest, the small amount of sample available requires the use of accelerator mass spectrometry (AMS) for radiocarbon analysis. A gram of modern carbon undergoes 13.56 radioactive disintegrations (^14^C→^12^C) per minute; this corresponds to a ^14^C concentration of 5.9 ×10^10^ atoms of ^14^C/gC. Because AMS detects atoms rather than radioactive decays, it is far more sensitive than conventional counting methods.

In AMS, a Cs^−^ ion sputter source is used to produce negative ions from a sample target. The major interference with ^14^C is ^14^N, which does not form stable negative ions and hence is largely eliminated. In many AMS systems, the mass-14 beam is selected by a magnetic deflector and directed into a tandem accelerator. The ions are accelerated at ~3 MeV and gas-stripped. At this energy, the most probable charge state for C is +3. Interfering molecular ions, e.g., ^13^CH and ^12^CH_2_, are unstable in the +3 state and disintegrate completely. The ion beam is then accelerated back to ground potential, the mass-14, +3 beam is selected, and final measurement of ^14^C occurs in E-d*E*/d*x* detectors of the type used in nuclear physics. Magnetic switching between ^14^C and ^13^C or ^12^C permits the measurement of ^14^C relative to a stable isotope.

At present, the accuracy of AMS is limited by contamination during sample preparation, sensitivity (response/^14^C) by ionization efficiency and halflife, and precision by counting statistics and “machine reproducibility” (currently ~0.5% for mg-size samples). A sample-preparation technique developed at NBS [8] involves a closed-tube combustion of samples to CO_2_ followed by a closed- system reduction to CO over hot Zn and reduction of CO to C on Fe wool. The procedure, giving an overall blank (chemistry and accelerator) of 1.9±0.4 μg contemporary carbon, has been used to measure samples containing <100 μg of carbon (RSE= 10%) [9].

Development of gaseous ion sources that can accept CO_2_ directly [10,11] will reduce sample handling and hence contamination. When such sources become available, combined techniques such as GC-AMS will be possible, since samples eluting from a column could be combusted to CO_2_ and introduced directly into the AMS ion source [12].

## 3. Radiocarbon in Atmospheric Particles

Carbonaceous particles in the atmosphere are of interest not only because of their effects on visibility, climate, and health but also because they may carry toxic or carcinogenic organic pollutants. Radiocarbon analysis allows modern particles arising from biological processes or from human activities such as wood-burning to be distinguished from those arising from fossil sources such as motor traffic and combustion of coal and oil.

Because of the large sample requirements of conventional decay counting, early work [13,14] involved collection of whole-particle samples over long periods (days). Such whole-particle samples are often biased by the presence of relatively large debris such as pollen, insect parts, and road dust, but the fine (<2.5 μm diameter) particles are of more environmental concern. Fine carbonaceous particles have a disproportionate effect on visibility and climate [15] and are in the “respirable” size range, i.e., they are retained in the lungs. Measurement of size-fractionated aerosol in Los Angeles [16,17,18] showed that the fine particles had a higher percentage of fossil, anthropogenic carbon than did larger particles.

Our involvement with EPA’s Integrated Air Cancer Project (IACP) [19] allowed us to investigate the sources of different chemical fractions in fine particles. Samples were collected during winter, 1984–1985, in two cities (Raleigh, NC, and Albuquerque, NM) where the dominant carbon sources were expected to be residential wood combustion (contemporary) and motor vehicle emissions (fossil). We used oxidation with nitric acid [20] to isolate the elemental carbon fraction from selected fine-particle samples and analyzed both elemental and total carbon for ^14^C. The results summarized in table 1 show that: (1) as expected, the fossil (motor vehicle) contribution was highest for the high-traffic intersection in the daytime and was quite low for the residential sites at night; and (2) the elemental carbon fraction had a higher fossil contribution than the corresponding total carbon in all cases. This result suggests that elemental carbon may be more useful than total or organic carbon for tracing mobile sources [21].

**Figure 1:**
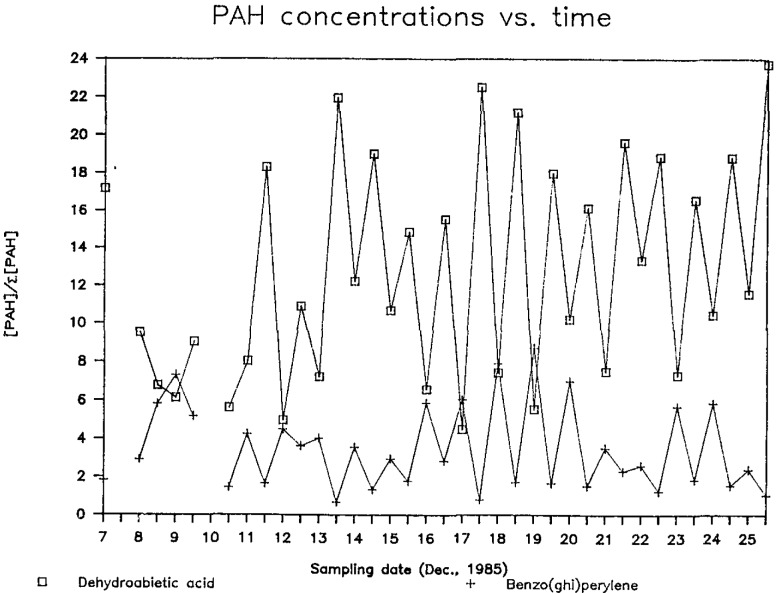
Variation of DHA and BGP with collection date and time in samples from a residential site in Albuquerque, NM. For each sample, PAH concentrations are expressed relative to the total mass of all PAH measured in that sample. Integral “date” values represent daytime samples; half-integral values indicate nighttime samples.

Finally, in a second Albuquerque study conducted in December 1985, we isolated the polycyclic aromatic hydrocarbon (PAH) fraction from fine-particle samples. The behavior of two PAH, dehydroabietic acid (DHA) and benzo(ghi)perylene (BGP), is shown in figure 1. DHA has been detected in wood smoke [22], and BGP has been suggested as a tracer for motor vehicle emissions [23]. The utility of these and other PAH as source tracers will be tested by comparing PAH-based source-strength estimates with ^14^C results for the PAH fraction.

## Figures and Tables

**Table 1:** Radiocarbon results for IACP samples

Site	% Contemporary	
	Total C	Elemental C
Raleigh, residential site		
night	95±14^a^ (3)^b^	60±11 (2)
Albuquerque, residential site		
night	78±6 (8)	61±10 (8)
day	66±2 (2)	40±18 (3)
Albuquerque, traffic intersection		
day	35±15 (3)	19±1 (2)

Contemporary carbon values have been adjusted for the ^14^C content of the atmosphere.

a Errors shown are standard deviations.

b The number of samples (*N*) for each entry is given in parentheses.
